# Improvement in the Oral Bioavailability and Efficacy of New Ezetimibe Formulations—Comparative Study of a Solid Dispersion and Different Micellar Systems

**DOI:** 10.3390/pharmaceutics12070617

**Published:** 2020-07-02

**Authors:** Carlos Torrado-Salmerón, Víctor Guarnizo-Herrero, Teresa Gallego-Arranz, Yvonne del Val-Sabugo, Guillermo Torrado, Javier Morales, Santiago Torrado-Santiago

**Affiliations:** 1 Department of Pharmaceutics and Food Technology, Faculty of Pharmacy, Complutense University, Plaza Ramón y Cajal s/n, 28040 Madrid, Spain; ctorrado@ucm.es (C.T.-S.); victor08@ucm.es (V.G.-H.); teresagallego_a@hotmail.com (T.G.-A.); ydelval@ucm.es (Y.d.V.-S.); 2 Department of Biomedical Science, Faculty of Pharmacy, University of Alcalá de Henares, Ctra Madrid-Barcelona Km 33,600, 28805 Madrid, Spain; guillermo.torrado@uah.es; 3 Department of Science and Pharmaceutical Technology, Faculty of Chemical and Pharmaceutical Sciences, University of Chile, Santiago 8380494, Chile; javiermv@ciq.uchile.cl; 4 Instituto Universitario de Farmacia Industrial, Complutense University, Plaza Ramón y Cajal s/n, 28040 Madrid, Spain

**Keywords:** ezetimibe, solid dispersion, micellar systems, enhanced dissolution, oral bioavailability, antihyperlipidemic effect

## Abstract

Ezetimibe (EZ) is a poorly water-soluble drug with low bioavailability. Strategies such as solid dispersions (SD) and micellar systems (MS) were developed to identify the most effective drug delivery formulations with the highest oral bioavailability, and to improve their lipid-lowering effect. The EZ formulations were prepared with different proportions of Kolliphor^®^ RH40 as a surfactant (1:0.25, 1:0.5 and 1:0.75) and croscarmellose as a hydrophilic carrier. These excipients, and the addition of microcrystalline cellulose during the production process, led to significant improvements in the dissolution profiles of MS. Powder X-ray diffraction (PXRD), differential scanning calorimetry (DSC) and scanning electron microscopy (SEM) revealed an amorphous form of ezetimibe with different semicrystalline states of microcrystalline cellulose for MS-I (1:0.75) and MS-II (1:0.75). Pharmacokinetic analysis after administration of MS-II (1:0.75) demonstrated a 173.86% increase in maximum plasma concentration (C_max_) and a 142.99% increase in oral bioavailability compared to EZ raw material (EZ-RM). Efficacy studies with the micellar system MS-II (1:0.75) in rats with hyperlipidemia showed that total cholesterol, triglycerides and high-density lipoprotein were reduced to normal levels and revealed improvements in low-density lipoprotein, aspartate and alanine aminotransferase. The improvement in the dissolution rate with micellar systems increases bioavailability and enhances the anti-hyperlipidemic effect of EZ.

## 1. Introduction

Ezetimibe is the first of a novel class of drugs used for the treatment of hyperlipidemia. This active pharmaceutical ingredient is characterized as class II in the Biopharmaceutics Classification System. Ezetimibe (EZ) is a poorly water-soluble drug with a low-dissolution profile, and its first-pass metabolism, enterohepatic recirculation and P-glycoprotein (P-gp) efflux also decrease the bioavailability of the drug [1].

Solid dispersions (SD) are one of the most widely used strategies to achieve an improvement in the dissolution of poorly soluble drugs, and SD with different hydrophilic polysaccharides such as hydroxypropyl methylcellulose (HPMC), hydroxypropyl cellulose (HPC) and croscarmellose have been used in recent years. The amphiphilic nature of these excipients improves the absorption of drug molecules within the polymer chains and reduces the thermodynamic instability of amorphous forms [2]. The addition of a surfactant with low ratios of hydrophilic carriers in SD enhances the formation of amorphous forms. The hydrophilic nature of the carrier may improve the dispersion of drug molecules with a lower proportion of polysaccharide chains [3].

Amphiphilic polymers and surfactants in a solid state are currently mixed with SD to prepare micellar systems by dispersion in aqueous media, and are known as ternary SD or micellar systems (MS) [3,4]. Surfactants such as sodium dodecyl sulphate, Tween 80 or Kolliphor^®^ RH40 have been used in MS to increase the dissolution profile of poorly soluble drugs [4]. The presence of surfactants in MS increases the molecular dispersion of the drug within the polymer chains, producing an amorphous form of the drug with low drug-to-polymer ratios between 1:1 and 1:3 [4]. The presence of a surfactant on the surface of the hydrophilic carrier reduces the surface tension of the MS and increases the dissolution profiles and bioavailability of the system [3].

EZ solid dispersions (SD) with low hydrophilic carrier ratios SD (1:1) and SD (1:2.5) usually exhibit very poor powder flow [5], possibly due to the small particle size of EZ. The addition of excipients such as microcrystalline cellulose or cellulose and silica can be used to increase the flowing properties of EZ [6,7]. The characterization of the polymer/surfactant interactions is especially important in MS formulations. Some characterization studies have been done on polymer/surfactant interactions with hydrophilic polymers such as Poloxamer, Soluplus^®^ and Eudragit^®^ [2,8]. However, very few studies have evaluated surfactant/polysaccharide interactions in MS formulations with carriers such as HPMC, HPC and croscarmellose [4,9].

Changes in crystallization during the formation of MS with different proportions of surfactant are evaluated with powder X-ray diffraction (PXRD) and differential scanning calorimetry (DSC) studies. The changes in melting temperatures and enthalpy values in the DSC studies were used to assess the influence of the surfactant on reducing drug crystallinity and on the polymer/polymer interactions of the carriers [3]. Moreover, microcrystalline cellulose (MC) can increase its degree of crystallinity during the elaboration process, changing the physicochemical properties of MC nanostructures [10].

Pharmacokinetic and pharmacodynamic studies have been performed on various hypolipidemic agents such as atorvastatine [4,11] and EZ [1,11,12]. Pharmacokinetic studies indicate that EZ metabolizes rapidly to a conjugated form (with high hypolipidemic activity) after oral administration. The total EZ in plasma has high concentrations (90%) of this conjugated form (EZ glucuronide) and low proportions (10%) of free-EZ [12]. The addition of β-glucuronidase to plasma samples is a method for analysing total EZ (free-EZ plus EZ glucuronide), which has been used to evaluate the bioavailability of EZ formulations in plasma and compare their antihyperlipidemic effects [1,12].

The aim of this study is to improve the solubility, dissolution and oral bioavailability of EZ using SD and MS formulations with different drug/surfactant ratios. In this work, two types of micellar systems with different manufacturing processes (MS-I and MS-II) were developed using croscarmellose and MC as carriers, and different ratios of Kolliphor^®^ RH40 as a surfactant. Characterization by PXRD, DSC and scanning electron microscopy (SEM) is important to indicate the decrease in EZ crystallinity and the polymer/polymer interactions for MS-I and MS-II formulations. These characterization studies allow the physicochemical properties of SD and MS formulations to be related with the results of the dissolution profiles. Efficacy studies in a hyperlipidemic rat model clarify the improvement in these micellar systems with rapid dissolution profiles and high oral bioavailability of EZ.

## 2. Materials and Methods

### 2.1. Materials

Ezetimibe (EZ) was obtained from Normon Pharmaceutical Co., Ltd. (Madrid, Spain). β-glucuronidase from bovine liver (>1,000,000 U/g) was purchased from Sigma (Sigma-Aldrich, Madrid, Spain). Croscarmellose sodium and microcrystalline cellulose (Avicel^®^ pH 101) were obtained from the FMC Corporation (Philadelphia, PA, USA). Sodium carboxymethyl cellulose (1000 cps) was procured from Sigma-Aldrich (Saint Louis, MO, USA). Kolliphor^®^ RH40 was purchased from the Basf Chemical Company (Barcelona, Spain). The water used in these studies was obtained from a Milli-Q water purification system (Billerica, MA, USA). All reagents and chemicals used were analytical grade.

### 2.2. Methods

#### 2.2.1. Preparation of Formulations

The ezetimibe raw material (EZ-RM) was used as reference in the dissolution, solubility, pharmacokinetic and biochemical studies. In DSC, SEM and PXRD, EZ-RM samples were used to characterize ezetimibe.

The physical mixture of EZ (PM 1:2.5) was prepared by mixing 100 mg of EZ and 250 mg of croscarmellose sodium in a ceramic bowl using a polymeric spatula, then adding 1000 mg of microcrystalline cellulose (MC) diluent and mixing.

A solid dispersion of EZ was prepared by dissolving 100 mg of EZ in 500 μL of ethanol by vortex (Fisherbrand^TM^; Milan, Italy) at 2500 rpm for 2 min. Two hundred and fifty milligrams of croscarmellose was then added to the EZ solution and mixed in a ceramic bowl using a polymeric spatula. The formulation was dried at 40 °C for 24 h and sieved between 0.297 and 0.850 mm. The microcrystalline cellulose (1000 mg) was added as a diluent after sieving and mixed with a polymeric spatula.

Micellar systems of EZ MS-I were prepared by dissolving 100 mg of EZ and different proportions of surfactant (Kolliphor^®^ RH40) in 500 μL of ethanol dissolved by vortex (Fisherbrand^TM^; Milan, Italy) at 2500 rpm for 2 min. The following amounts of surfactant were added to the MS-I formulations: 25 mg for MS-I (1:0.25), 50 mg for MS-I (1:0.5) and 75 mg for MS-I (1:0.75). Each solution of EZ and Kolliphor^®^ RH40 in ethanol was added to a ceramic bowl with 250 mg of croscarmellose and 1000 mg of MC and mixed using a polymeric spatula. The micellar systems were then dried at 40 °C for 24 h. The formulations were sieved to isolate the 0.297–0.850 mm fraction.

Micellar systems of EZ MS-II were prepared with 100 mg of EZ and different amounts of Kolliphor^®^ RH 40 for each MS-II formulation: 25 mg for MS-II (1:0.25), 50 mg for MS-II (1:0.5) and 75 mg for MS-II (1:0.75). These proportions of EZ:Kolliphor^®^ RH 40 were dissolved in 500 μL of ethanol using a vortex (Fisherbrand^TM^; Milan, Italy) at 2500 rpm for 2 min. The solutions of EZ and Kolliphor^®^ RH40 in ethanol were mixed in a ceramic bowl with 250 mg of croscarmellose. The formulations were dried at 40 °C for 24 h and then sieved between 0.297 and 0.850 mm. The microcrystalline cellulose diluent (1000 mg) was then added to each formulation and mixed with a polymeric spatula.

#### 2.2.2. Flow Properties

Flow properties were studied by measuring the angle of repose (°), Carr’s index (%) and Hausner ratio [6]. The angle of repose of the different formulations was determined by the fixed funnel method. The physical mixtures of each formulation were accurately weighed and placed in a funnel set to a height of 2.5 cm. The powder was allowed to flow and the height and diameter of the powder cone were measured; the angle of repose was then calculated according to the following formulation:

tan θ=2hd

In this formulation θ is the angle of repose, h is the height of the cone in cm and d is the diameter of the cone base in cm.

Carr’s index (CI) of each sample was determined with the formulation:

$$\mathrm{CI}=\frac{D_{t\;}{-\;D}_{b}\;}{D_{t}}$$

In this formulation D_t_ is the tapped density (g/cm^3^) of the powder until no more change in sample volume was noticed (100 taps) and D_b_ is the bulk density (g/cm^3^) calculated using the sample total volume (includes particle volume and inter-particle void volume).

The Hausner ratio (H) was calculated using the tapped density (D_t_) and the bulk density (D_b_):

H= DtDb

#### 2.2.3. Solubility Study

Solubility studies were performed using excess amounts of each formulation, which were added to test tubes containing 3 mL of acetate buffer 0.05 M with a pH of 4.5. The tubes were mixed in a vortex and placed in a shaking water bath at 37 °C for five days, then analyzed by a spectrophotometric method [13]. Samples were filtered through a 0.45 μm filter (Acrodisc^®^, Port Washington, NY, USA). The cumulative amount of EZ released from the formulations was determined at 233 nm with a UV-VIS JASCO V-730 spectrophotometer (Jasco^®^ International Co. Ltd.; Tokyo, Japan), with the following calibration curve: *y* = 0.0394 × (μg/mL) − 0.0089 (r^2^ = 0.9996) across a range of 1–15 μg/mL. Each determination at each time was performed in triplicate, and the error bars on the graphs represent the standard deviation.

#### 2.2.4. Dissolution Study

The dissolution studies were performed using the United States Pharmacopeia (USP) paddle method (apparatus 2) in ERWEKA DT 80 (ERWEKA GmbH; Langen, Germany) dissolution equipment with a rotational speed of 50 rpm and 500 mL of dissolution medium (0.05 M sodium acetate buffer) adjusted to pH 4.5 (USP42-MF37, 2019), and containing 0.45% of sodium lauryl sulphate. The temperature was maintained at 37.0 °C ± 0.5 °C throughout the dissolution study. Various formulations of the micellar systems MS-I and MS-II with an amount equivalent to 10 mg of EZ were placed in the dissolution vessels, and 10 mg of EZ-RM was used as the EZ reference. Samples were collected at different times and filtered through a 0.45 μm filter (Acrodisc^®^, Port Washington, NY, USA). The EZ released from the formulations was determined at 233 nm by the spectrophotometric method described in the solubility study. Each determination at each time was performed in triplicate and the error bars on the graphs represent the standard deviation.

#### 2.2.5. Powder X-ray Diffraction (PXRD)

PXRD patterns were recorded on a Philips X’Pert-MPD X-ray diffractometer (Malvern Panalytical; Almelo, The Netherlands) in the CAI (Centro de Asistencia a la Investigación, Universidad Complutense de Madrid, Madrid, Spain) XRD technological research facility. The samples were radiated using a monochromatized CuKα radiation (λ = 1.542 Å), then analyzed between the 5 and 40° (2θ) range at a step size of 0.04° and a time of 1 s per step. The voltage was 30 kV with a current of 30 mA. The crystallinity index (CrI) of MC was calculated using the peak intensity method [14]:

$$\mathrm{CrI}=\;\frac{I_{2\theta }{\;-\;I}_{\mathrm{am}}}{I_{2\theta }}\text{×}100$$

In this equation I_2θ_ is the intensity of the peak at 2θ = 22.5° and I_am_ is the minimum intensity corresponding to the amorphous content at 2θ = 18°.

#### 2.2.6. Differential Scanning Calorimetry (DSC)

Samples were mounted on a Star system TC 15 TA double-sided automatic thermal analyzer (Mettler Toledo; Schwerzen-bach, Switzerland). The temperature was calibrated using the Indium Calibration Reference Standard with a transition point of 156.60 °C. All the samples were accurately weighed into aluminium pans and hermetically sealed with aluminium lids, then heated from 0 °C to 240 °C at a rate of 10 °C/min under constant purging of dry nitrogen at 20 mL/min. An empty pan was sealed and used as a reference with the same sample conditions. The heat of fusion of each formulation in the DSC results was used to calculate the degree of EZ crystallinity (%) according to the following equation [15]:

$$\mathrm{Xc}=\frac{{\Delta H}_{m}}{{\Delta H^{{}^{\circ}}}_{m}\;(W)}\text{×}100$$

where Xc is the degree of EZ crystallinity, ΔH_m_ is the heat of fusion of the sample tested, ΔH°_m_ is the heat of fusion of the EZ 100% crystalline form (heat of fusion of EZ-RM) and W is the fraction of the weight of the samples tested.

#### 2.2.7. Scanning Electron Microscopy (SEM)

Samples were mounted on double-sided adhesive tape and sputtered under vacuum with a thin gold–palladium layer using a Q150RS–rotary pumped coater (Quorum technologies; Laughton, UK). The samples were then analyzed with a Jeol JSM-6400 (Jeol Ltd.; Peabody, MA, USA) scanning electron microscope at an acceleration voltage of 20 kV by energy-dispersive X-ray microanalysis. All micrographs were the product of secondary electron imaging used for surface morphology identification at a magnification of 1000×.

#### 2.2.8. Animal Study

The experimental procedure was conducted with 18 male Wistar rats for the pharmacokinetic studies and 24 male Wistar rats for the total cholesterol and lipid profile studies. The animals were supplied by Envigo Rms Co., Ltd. (Barcelona, Spain), with an average weight of 242.64 ± 20.06 g. This animal experiment was carried out in the Animal Experimentation Centre at the University of Alcalá de Henares. The study was approved with project identification code ES280050001165, following the regulations of the University’s Ethics Committee, Madrid Region PROEX 041/18 (4/27/2018). The animals were housed in standard cages with unrestricted access to food and water throughout the experiment, in 12-h light–dark cycles and in a temperature-controlled animal room.

#### 2.2.9. Pharmacokinetic Study

##### Plasma Concentration Profile

Pharmacokinetic experiments were performed on three groups of animals (n = 6): EZ-RM, MS-I (1:0.75) and MS-II (1:0.75). Animals were fasted overnight before the experiment. The formulations were suspended in a sodium carboxymethyl cellulose solution (0.75% *w/v*), and 0.4 mL of each suspension, equivalent to 3 mg/Kg of EZ, was administered to the rats through oral gavage. Blood samples were collected from the tails of the rats at 0.5, 1, 1.5, 2, 4, 6, 8 and 24 h. The samples were transferred to heparinized Eppendorf tubes and centrifuged at 9000 rpm for 15 min to collect the plasma. The plasma homogenates were stored at −20 °C.

For EZ plasma quantification, 100 µL of plasma was transferred to Eppendorf test tubes and 50 µL of β-glucuronidase (>100,000 IU/g) was added to each tube. After vortexing for 2 min, the tubes were incubated at 50 °C for 1 h. To precipitate the proteins, 400 μL of acetonitrile was added to each sample. The Eppendorf tubes were stirred for 2 min at 2500 rpm and centrifuged at 9000 rpm for 10 min, and the supernatant was filtered through a 0.45 µm Millipore filter (Acrodisc^®^, Port Washington, NY, USA).

High-performance liquid chromatography (HPLC) (Agilent 1100 series FLD G1321A) equipped with a 50 µL loop was used to quantify EZ in plasma. Samples were separated using a Zorbax SB C-8 column (250 × 4.6 mm, 5 mm). The mobile phase consisted of acetate buffer pH 4.0 (40%) and HPLC-grade acetonitrile (60%). This mobile phase was degasified by placing in an ultrasonic bath for 5 min and filtered at 0.45 µm. The flow rate of the mobile phase used in the HPLC was 1 mL/min, and the EZ was analyzed at a wavelength of 233 nm.

The calibration curve (y = 16,111 x + 1152.70), (r^2^ = 0.9996) was performed by adding 20 µL of different concentrations of EZ with 80 µL of blank rat plasma (n = 9). The method had a concentration range of 1.0–30.0 ng/mL, with a limit of detection of 0.3 ng/mL and a limit of quantification of 1.0 ng/mL. An itraconazole solution was used as an internal standard. This method was validated according to International Conference on Harmonization (ICH) Q2 (R1) (Committee for Proprietary Medicinal Products (CPMP/ICH/381/95).

##### Bioavailability Parameters

Pharmacokinetic parameters were estimated for individual rats in each group from plasma concentration time curve data within 24 h of drug intake using pharmacokinetic functions [5]. The areas under curves (AUC_0–24_) were calculated by means of the trapezoidal rule. The maximum plasma concentration (C_max_) and time to reach peak plasma concentration (T_max_) for each formulation (EZ-RM, MS-I and MS-II) were estimated as the mean values of the six animals used per group with standard deviation. Relative bioavailability was calculated by dividing the AUC_0–24_ of the test samples with the EZ raw material (EZ-RM) as reference sample. Comparative statistical studies on the bioavailability of the different formulations were done using Tukey’s test, with a one-way ANOVA test in the Statgraphics program (Statgraphics Technologies, The Plains, VA, USA).

##### Lipid Profile Analysis

The biochemical parameters were studied in four groups of animals (n = 6): control group, high-fat diet (HFD) group, EZ-RM group and MS-II (1:0.75) group. For the studies of lipid profiles in rats with induced hyperlipidemia, the animals were fed a high-fat diet (HFD) composed of fats and cholesterol (18 g of fat and 2 g of cholesterol per 100 g of diet) administered during eight weeks before the beginning of the treatment with EZ. The control group was fed a standard diet. At the end of the eight weeks, the serum lipid profiles were measured.

The treatments with the EZ-RM and MS-II formulation (1:0.75), equivalent to a 3 mg/kg dose of EZ, were suspended in sodium carboxymethyl cellulose (0.75% *w/v*), and 0.4 mL was administered to the rats through oral gavage. The control group and the HFD group received only 0.4 mL of sodium carboxymethyl cellulose (0.75% *w/v*). After two and four weeks, blood samples were collected from the rats’ tails in Eppendorf tubes for biochemical analysis. The animals were fasted overnight before each blood draw.

The serum was separated by centrifugation at 4000 rpm for 10 min (M12P centrifuge; Neuation Technologies, Gandhinagar, India). Serum concentrations of total cholesterol (TC), triglycerides (TG), high-density lipoprotein (HDL), aspartate transaminase (AST) and alanine transaminase (ALT) were measured using commercial diagnostic kits (Biosystems Barcelona, Spain; Spinreact Gerona, Spain). The low-density lipoprotein (LDL) concentrations were calculated using the Friedewald equation. The data were represented as mean mg/dL ± standard deviation. The effect of hypercholesterolemia in the rat model was evaluated by Tukey’s test, with a one-way ANOVA test using Statgraphics (Statgraphics Technologies, The Plains, VA, USA).

## 3. Results and Discussion

### 3.1. Flow Properties

Powder mixtures must be evaluated to determine the dosage of the formulations using standardized parameters, such as the angle of repose (°) and Carr’s index, that are widely used by the pharmaceutical industry [6].

EZ raw material (EZ-RM) had an angle of repose of >40°, a Carr’s index of 35.51% ± 2.92% and a Hausner ratio of 1.55 ± 0.07, indicating poor fluidity and compressibility (Table 1) owing to the small particle size of the EZ. The solid dispersion of EZ (SD) showed an improvement in the angle of repose compared to EZ-RM, but presented poor values for the Carr’s index and Hausner ratio. The micellar systems MS-I (1:0.75) and MS-II (1:0.75) showed poor flowability, although with a slight improvement in the angle of repose, which was probably due to the presence of the croscarmellose carrier (Table 1). Poor powder flow properties in solid dispersions of the drug with a small particle size have been observed previously [7].

High percentages of microcrystalline cellulose (MC) are needed to improve the flow properties of the EZ formulations. The SD showed significant improvements (*p* < 0.05) in the angle of repose, Carr’s index and Hausner ratio for the EZ/MC (10/100) proportions compared to the 10/50 EZ/MC ratio. The MS-I and MS-II micellar systems with different manufacturing processes showed slight improvements in flow properties. The results for the different EZ: Kolliphor^®^ RH40 ratios 1:0.25, 1:0.5 and 1:0.75 exhibited no significant differences (*p* > 0.05) for both MS-I and MS-II micellar systems. The formulations MS-I (1:0.75) and MS-II (1:0.75) with the 10/50 EZ/MC ratio had slight improvements in flow properties. However, the 10/100 EZ/MC ratio showed good results with angles of repose of 22.67 ± 2.31° and 17.67 ± 2.08° for MS-I (1:0.75) and MS-II (1:0.75), respectively. A similar improvement was observed for Carr’s index and Hausner ratio in both MS-I and MS-II micellar systems with different manufacturing processes. These results suggest that MS-I and MS-II with 10/100 EZ/MC ratios are necessary to improve the flow properties of EZ formulations [7]. Similar amounts of excipients have previously been added to an ultra-fine dispersion of EZ obtained by spray-drying to obtain a free-flowing powder and prevent the agglomeration of EZ particles [6].

### 3.2. Solubility Study

The low solubility of EZ in simulated intestinal medium at pH 4.5 (1.99 ± 0.24 µg/mL) requires a combination of different technological resources to increase its solubility. The physical mixture of EZ (PM) showed a slight improvement in solubility, and the solid dispersions of EZ (SD) presented 2.63-fold increases compared to EZ-RM. This improvement in SD was related to the water absorption and swelling properties of the croscarmellose and MC, which favored the dispersion and wettability of the EZ. The drug-carrier interactions in the solid dispersion (e.g., hydrogen bonding) and its wettability could explain these increases in the solubility of EZ [4].

A microscopic study of the residual precipitate at the end of the solubility studies showed an agglomeration of EZ crystals for EZ-RM and PM samples. However, the SD formulation showed less agglomeration of EZ particles with smaller crystals. These results confirmed an increase in the amorphous form of EZ present in SD which improves solubility. Similar increases in the solubility of poorly soluble drugs have been observed with various hydrophilic cellulose polymers [13,16].

**Figure 1:**
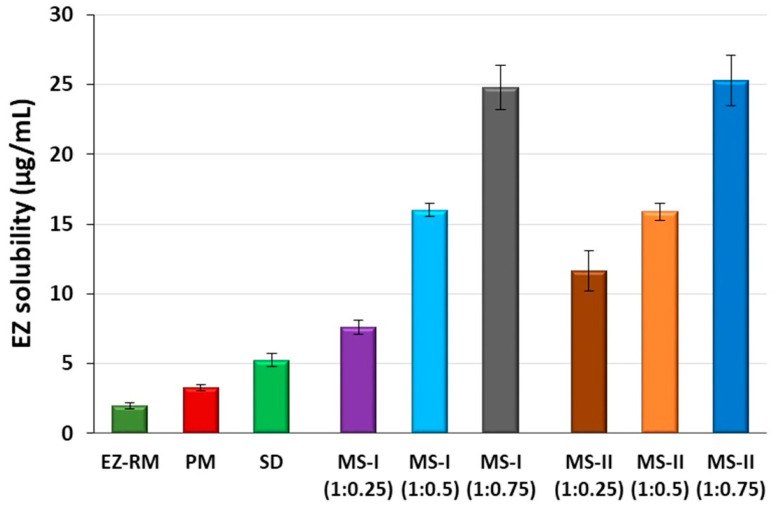
Solubility concentrations of EZ raw material (EZ-RM), physical mixture (PM), solid dispersion (SD) and micellar systems with Kolliphor^®^ RH40: MS-I (1:0.25), MS-I (1:0.5), MS-I (1:0.75), MS-II (1:0.25), MS-II (1:0.5) and MS-II (1:0.75) at pH 4.5.

The micellar systems with Kolliphor^®^ RH40 MS-I and MS-II showed improvements in the solubility studies. These formulations presented higher solubility values compared to SD (Figure 1). The use of Kolliphor^®^ RH40 as a surfactant significantly improves the solubility of EZ with 3.82 and 5.85-fold increases for MS-I (1:0.25) and MS-II (1:0.25), respectively, compared to EZ-RM. The 1:0.75 EZ:surfactant ratio produced the highest EZ solubility values, with similar 12.46 and 12.72-fold increases for MS-I (1:0.75) and MS-II (1:0.75), respectively, compared to EZ-RM.

The solubility results with SD and micellar systems with low surfactant ratios MS-I (1:0.25) and MS-II (1:0.25) produced a fast precipitation process related to slower micelle formation at pH 4.5. The solubility differences between MS-I (1:0.25) and MS-II (1:0.25) may be related to the different elaboration process for MS-I and MS-II. However, there was no difference between MS-I and MS-II with the ratios 1:0.5 and 1:0.75. The high solubility results observed with the highest surfactant ratios in MS-I (1:0.75) and MS-II (1:0.75) indicated a more rapid micelle formation process at this pH [17]. The differences between the formulations with ratios 1:0.25, 1:0.5 and 1:0.75 may be related to an improvement in the interaction of the surfactant with the croscarmellose chains and ezetimibe particles, MS-I (1:0.25) may present a slower micelle formation process and increase EZ precipitation, thus reducing its solubility values [16]. The microscopic study of the residual solids of these formulations at the end of the solubility showed significant decrease (*p* < 0.05) in EZ crystalline precipitate with a lower amount of agglomerated particles for MS-I (1:0.75) and MS-II (1:0.75) compared to MS-I (1:0.25) and MS-II (1:0.25).

### 3.3. In Vitro Drug Release

**Figure 2:**
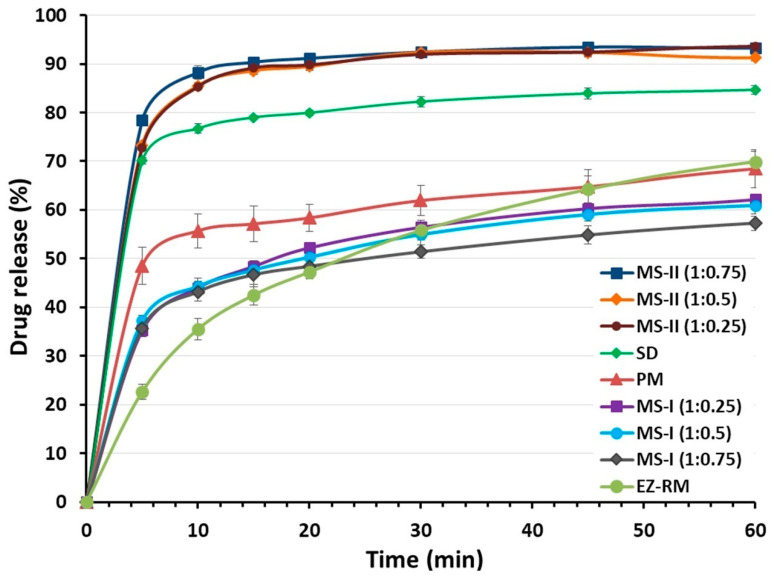
Dissolution profiles of EZ raw material (EZ-RM), physical mixture (PM), solid dispersion (SD) and micellar systems with Kolliphor^®^ RH40: MS-I (1:0.25), MS-I (1:0.5), MS-I (1:0.75), MS-II (1:0.25), MS-II (1:0.5) and MS-II (1:0.75).

EZ-RM had a slow dissolution profile in these studies, with 35.54% ± 1.55% and 64.23% ± 2.70% at 10 and 45 min respectively (Figure 2). Similar slow dissolution profiles for EZ have previously been reported [5]. The dissolution rate of the EZ physical mixture (PM 1:2.5) with the addition of MC showed a significant 1.57-fold increase (*p* < 0.05) at 10 min and a similar percentage of dissolution at 45 min (64.76% ± 3.58%) compared to EZ-RM. The presence of hydrophilic excipients such as croscarmellose and microcrystalline cellulose improves wettability and reduces the interfacial tension between EZ and the dissolution medium, resulting in a relatively higher dissolution rate [3]. The solid dispersion (SD) produces a significant improvement in dissolution profiles during the initial times with a significant 2.16-fold increase (*p* < 0.05) at 10 min and a 1.31-fold increase at 45 min, compared to EZ-RM. SD showed higher dissolution profiles compared to MS-I formulations, but lower profiles than MS-II micellar systems. The different manufacturing process of the MS-I formulations increases recrystallization and decreases the dissolution profiles. The presence of a higher amount of EZ’s amorphous form in SD and the incorporation of the hydrophilic excipient MC improves wettability and increases its dissolution profiles.

Ternary solid dispersions MS-I (1:0.25), MS-I (1:0.5) and MS-I (1:0.75) had similar dissolution percentages (44.02% ± 1.26%, 44.32% ± 1.69% and 43.21% ± 1.94%, respectively) at 10 min. The MS-I formulations had a lower dissolution profile than the PM (1:2.5), although their dissolution rates were higher than EZ-RM (Figure 2). It is important to note that the dissolution profile in the micellar systems MS-I (1:0.25), (1:0.5) and (1:0.75) does not change as the surfactant ratio increases. The addition of microcrystalline cellulose (MC) before the drying process produces a partial dispersion of the EZ within the MC, and a slow diffusion out of the matrix was observed [18].

The micellar systems of EZ MS-II showed a significant increase in dissolution profiles compared to the other EZ formulations (Figure 2). The similar dissolution rates of MS-II (1:0.25) and MS-II (1:0.5) may be due to the formation of a submicellar structure [3]. A previous study showed that submicellar or micellar systems of Kolliphor^®^ RH40 increase the solubility of poorly soluble drugs [9]. However, the micellar system MS-II (1:0.75) showed a slight increase in dissolution percentages compared to MS-II (1:0.25) and MS-II (1:0.5), which may be related to the significant improvement observed in the solubility studies (Figure 1). The MS-II (1:0.75) formulation also showed a significant 2.48-fold increase (*p* < 0.05) at 10 min and a 1.46-fold increase at 45 min compared to EZ-RM. The increase in the ratio of Kolliphor^®^ RH40 enhances the mobility of the amorphous form of EZ, and improves the formation of micellar systems and the solubility of EZ at pH 4.5. This may be due to the presence of surfactant on the surface of the hydrophobic EZ particles that could reduce their aggregation during dissolution [5].

### 3.4. Powder X-ray Diffraction (PXRD)

**Figure 3:**
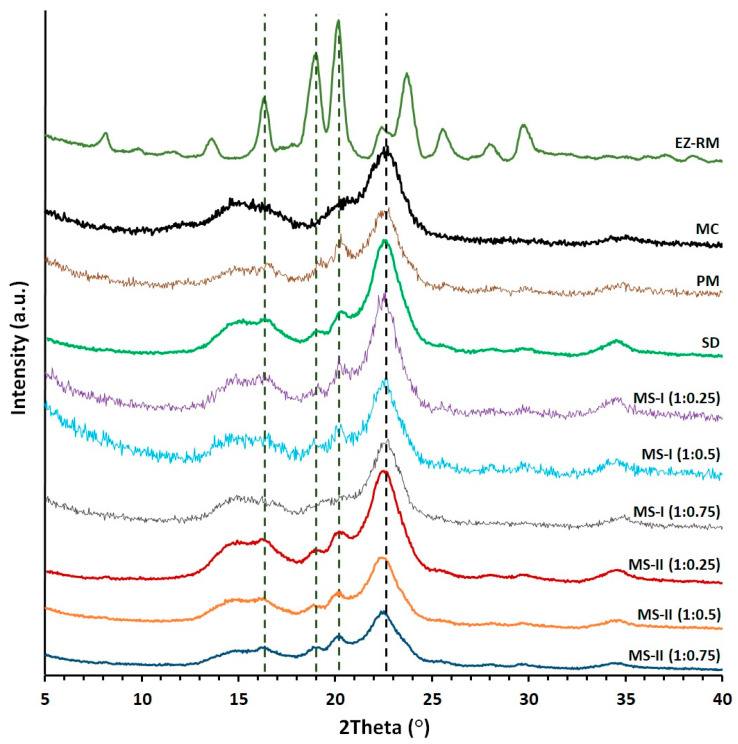
Powder X-ray diffraction (PXRD) patterns of EZ raw material (EZ-RM), microcrystalline cellulose (MC), physical mixture (PM), solid dispersion (SD) and micellar systems with Kolliphor^®^ RH40: MS-I (1:0.25), MS-I (1:0.5) and MS-I (1:0.75), MS-II (1:0.25), MS-II (1:0.5) and MS-II (1:0.75).

PXRD studies are used to determine changes in the crystallinity of EZ and MC for the micellar systems MS-I and MS-II. Figure 3 compares the X-ray diffraction patterns of EZ-RM, PM and formulations SD, MS-I and MS-II.

EZ-RM shows sharp peaks corresponding to the crystal structure with major diffraction angles at 16.32 °, 18.93 °, 20.11° and 23.69° 2θ (Figure 3), related to anhydrous EZ [19]. PXRD patterns of MC show two semi-crystalline halos between 13–18° and 18–25° 2θ, and the croscarmellose presented a semi-crystalline halo with a diffraction intensity between 16–25° 2θ [9]. A previous study with different proportions of EZ/croscarmellose and EZ/MC allowed us to select peaks at 16.32 °, 18.96° and 20.11° 2θ to analyze the decrease in the crystallinity of the EZ in the different formulations. The comparison of MC with PM and SD formulations in Figure 3 showed MC halos between 13–18° and 18–21° 2θ with a low diffraction intensity of the MC, which did not modify the intensity of the EZ peaks. PM showed characteristic diffraction peaks of EZ at 16.32 °, 18.96° and 20.17° 2θ. The study of EZ at 18.96° and 20.17° 2θ showed a decrease in crystallinity index as a result of a dilution effect of the croscarmellose.

The SD presented a decrease in diffraction intensity peaks at 16.33°, 18.94° and 20.19° 2θ. The EZ patterns showed significant decreases in crystallinity compared to EZ-RM. The preparation of an SD formulation improves the interaction between EZ molecules and croscarmellose chains, which increases the amorphous form of EZ. The MC did not show changes in its crystallinity during its elaboration process. MS-I ternary solid dispersions revealed significant changes in diffraction patterns for EZ and MC. These micellar systems showed decreases in intensity in the EZ peaks at 18.93° and 20.11° 2θ respectively. The MS-I formulations (1:0.75) presented a decrease in intensity peaks related to the higher percentages of Kolliphor^®^ RH40. Lower drug crystallinity has been observed previously in a solid dispersion with a surfactant [10]. Moreover, all the MS-I micellar systems had a broad halo with a higher crystalline shape between 21–25° 2θ, corresponding to the semi-crystalline MC halo. The MC showed a significant increase in crystallinity index during the MS-I preparation. This change was attributed to a significant surfactant/polymer interaction.

The ternary solid dispersion with the addition of MC after the drying process, MS-II (1:0.25), showed a decrease in the intensity peaks of the EZ at 18.96° and 20.17° 2θ compared to MS-I (1:0.25). This micellar system also showed no changes in the crystallinity index of the MC halo (18–25° 2θ) compared to the SD formulation. These results indicated that the MS-II process is more suitable for decreasing the crystallinity of the EZ without recrystallizing the MC in the formulation. Several studies have attributed a decrease in crystallinity in PXRD to similar surfactant interactions with different excipients [10].

MS-II (1:0.5) and MS-II (1:0.75) showed the greatest decreases in diffraction patterns for peaks at 18.93° and 20.11° 2θ. These results are related to a high proportion of the amorphous form of EZ. The presence of high proportions of Kolliphor^®^ RH40 during the preparation process of the MS-II improved the inclusion of EZ molecules in the croscarmellose chains and decreased the EZ crystallinity. Amorphous forms of EZ in surfactant systems have been observed previously [2]. All the MS-II micellar systems exhibited a semi-crystalline halo (21–25° 2θ) of MC, which had a similar or lower crystalline shape compared to the MC raw material, indicating that the addition of MC after the drying process avoided the surfactant/polymer interaction observed in MS-I.

### 3.5. Differential Scanning Calorimetry (DSC)

**Figure 4:**
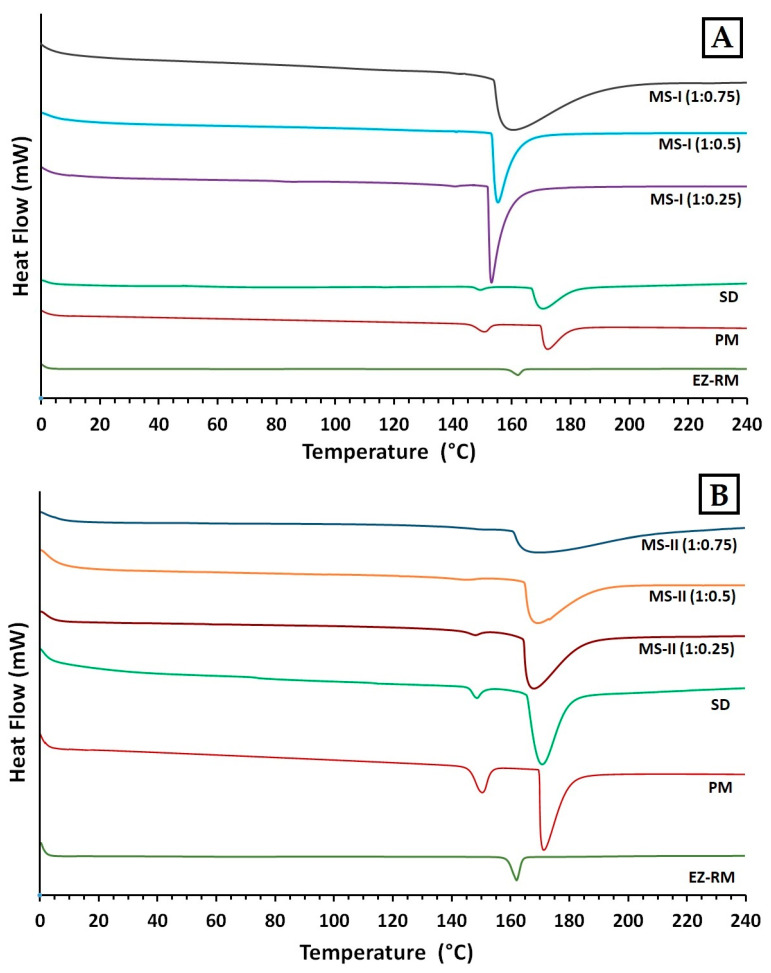
Differential scanning calorimetry (DSC) thermograms of EZ. (**A**): EZ raw material (EZ-RM), physical mixture (PM), solid dispersion (SD) and micellar systems with Kolliphor^®^ RH40: MS-I (1:0.25), MS-I (1:0.5) and MS-I (1:0.75). (**B**): EZ raw material (EZ-RM), physical mixture (PM), solid dispersion (SD) and micellar systems with Kolliphor^®^ RH40 MS-II (1:0.25), MS-II (1:0.5) and MS-II (1:0.75).

Figure 4 shows the differences in DSC curves between EZ raw material (EZ-RM), physical mixture (PM), solid dispersions (SD) and two types of micellar systems with Kolliphor^®^ RH40 (MS-I and MS-II).

The EZ raw material (EZ-RM) presented a sharp endothermic peak at 162.24 °C with an enthalpy of fusion of 77.02 J/g (Figure 4B) [13]. The MC showed a broad endothermic peak at 183.66 °C. Physical mixtures (PM) showed lower melting temperatures in the first endothermic peak (150.34 °C) corresponding to the interaction of EZ/croscarmellose, and a second broad endothermic peak at 173.16 °C due to the croscarmellose/MC interaction (Figure 4A). The croscarmellose chains facilitate the mobility of the EZ molecules and reduce the crystallinity of the first endothermic peak [9]. The temperature increase in the second endothermic peak of PM confirms that croscarmellose chains interact with MC chains.

The SD formulation showed a decrease in the first endothermic peak at 148.73 °C with an EZ crystallinity around 22%. The presence of this amount of the amorphous form of EZ is related with the decrease observed in PXRD, and could explain the increase in the EZ dissolution profile. This solid dispersion showed a second peak at 171.03 °C, which indicates an interaction between the croscarmellose and MC. The micellar system MS-I (1:0.25) showed a decrease in the temperature of the endothermic peak of EZ/croscarmellose (142.03 °C) and a crystallinity around 14%, corresponding to an EZ interaction with the croscarmellose (Figure 4A). The second endothermic peak of MS-I (1:0.25) at 153.76 °C showed a significant increase in the crystallinity of MC compared to PM and SD formulations, which may be due to the addition of MC before the drying process. The crystallized MC hinders the interaction between the EZ and the surfactant. Similar effects on chain mobility have been observed with other cellulose derivatives with surfactants [3].

The first endothermic peak in the formulations MS-I (1:0.5) and MS-I (1:0.75) presented significant decreases in the crystallinity of the EZ (13% and 12% respectively), and an increase in the temperatures of the endothermic peaks of MC (155.58 °C and 160.13 °C respectively) in comparison with MS-I (1:0.25) (Figure 4A). The absence of a first endothermic peak in Figure 4A indicates the presence of EZ in a practically amorphous form. These first endothermic peaks at 143.11 °C and 142.79 °C could only be observed after magnification (20 mW heat flow). These results suggest that a greater surfactant ratio increases the surface tension and mobility of EZ molecules and the amorphous form of EZ. Significant decreases were observed in the crystallinity of the second endothermic peaks for MS-I (1:0.5) and MS-I (1:0.75), respectively. High surfactant ratios improve the mobility of MC chains and decrease the crystallinity of the MC. The partial recrystallization of MC observed in the PXRD and DSC studies for MS-I (1:0.25), MS-I (1:0.5) and MS-I (1:0.75) may be due to the low dissolution profiles of these formulations. A similar result was previously described in MC granules containing irbersartan nanocrystals [20].

In DSC studies, the MS-II micellar systems showed a significant decrease in crystallinity compared to the MS-I formulations. MS-II (1:0.25) showed a first endothermic peak at 147.50 °C (Figure 4B) with a low crystallinity percentage around 10%, caused by the EZ/croscarmellose interactions. The MS-II (1:0.25) also exhibited a second endothermic peak at 167.53 °C for the MC/croscarmellose chains. The high decrease in EZ crystallinity in MS-II was related to an increased mobility of EZ molecules within the croscarmellose carrier, improved by the surfactant interaction. These results agree with the partial amorphization of EZ observed in PXRD. Similar decreases in the crystallinity of EZ have been obtained in micro and nanoparticles of poorly soluble drugs [21].

The thermographs of the MS-II (1:0.5) and MS-II (1:0.75) micellar systems did not show the first endothermic peak of the EZ in Figure 4B. After a magnification of these thermographs (5 mW heat flow), this first endothermic peak could be observed at 145.53 °C and 146.79 °C, respectively, with the lowest EZ crystallinity percentages for both micellar systems (around 6% and 3% respectively). One possible explanation for the amorphous form of EZ could be the high surfactant ratios, which facilitate the dispersion and mobility of croscarmellose chains, favouring stronger intermolecular interactions between EZ/croscarmellose and inhibiting the recrystallization of EZ [22]. MS-II (1:0.5) and MS-II (1:0.75) also presented a second broad endothermic peak at 168.31 °C and 166.97 °C. The low crystallinity percentages were due to the increased mobility of the MC/croscarmellose chains. The development of amorphous forms of EZ and the decrease in carrier crystallinity in MS-II (1:0.75) led to a significant improvement in EZ dissolution. Similar improvements in dissolution profiles were obtained by developing formulations containing the amorphous form of the different drugs produced [5,16].

### 3.6. SEM Characterization

**Figure 5:**
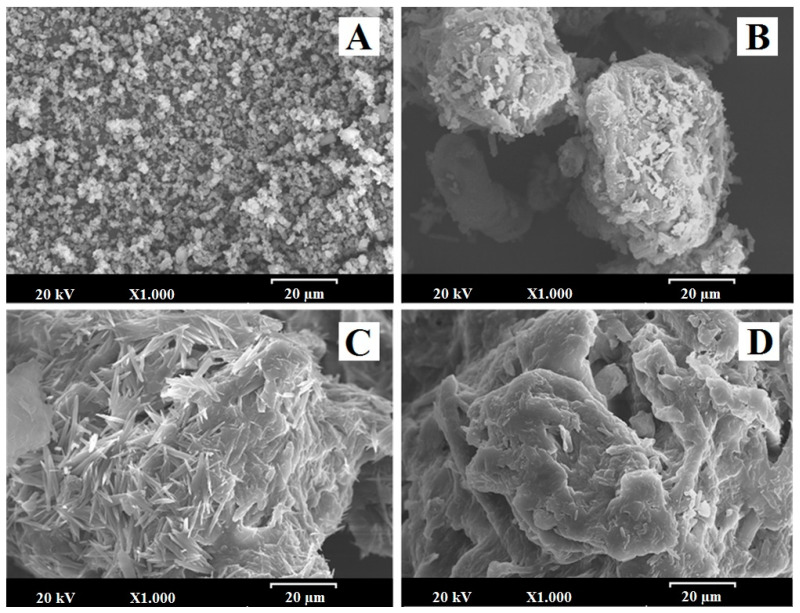
SEM micrographs of (**A**) EZ raw material (EZ-RM), (**B**) physical mixture (PM), (**C**) micellar system MS-I (1:0.75) and (**D**) micellar system MS-II (1:0.75). Photographs were taken at a magnification of 1000×.

Figure 5 shows the microphotographs of EZ raw material (EZ-RM), physical mixture (PM-EZ) and micellar systems MS-I (1:0.75) and MS-II (1:0.75). Small spherical particles of EZ-RM with a size between 0.5–2 µm can be seen in Figure 5A, which also shows some aggregations of the EZ particles. This morphology is common in hydrophobic drug microparticles and was previously observed in other studies [4,13].

The PM (1:2.5) had large irregularly-shaped particles (between 50 and 75 µm) formed by the aggregation of croscarmellose and MC fibres (Figure 5B). This SEM image reveals small crystals of EZ on the surface of the croscarmellose and MC aggregations.

Significant differences in particle morphology are observed depending on the preparation process. The ternary solid dispersion MS-I (1:0.75) shows a matrix structure corresponding to the aggregation of the croscarmellose carrier with the MC fibres, with a film of surfactant on the surface (Figure 5C). Almost none of the small EZ crystals were observed on the surface of the carrier. However, a new morphological structure can be seen during the MS-I formation process, with crystals between 10 and 20 µm caused by the partial crystallization of MC during the drying process. This matrix structure could be related to the sustained release profile observed in dissolution studies. The partial crystallization of the MC was confirmed by the increase in its endothermic peak in MS-I (DSC studies). The presence of crystalline particles on the surface of micellar systems has been described previously [4,23].

The micellar systems show major changes on the surface of the croscarmellose and MC aggregation. MS-II (1:0.75) revealed a homogeneous film of surfactant on the surface of the croscarmellose with the EZ particles trapped inside (Figure 5D). EZ molecules were identified in different samples by energy-dispersive X-ray microanalysis [24]. The interaction enhancement between EZ and croscarmellose by the addition of the surfactant has been shown in these SEM studies. The elasticity of the surfactant film improves the aggregation process between the large elongated MC fibres and the croscarmellose carrier particles. The absence of small acicular MC crystals was attributed to the addition of the MC after the drying process. These results may be related to the decrease in the semicrystalline peak observed in the PXRD and DSC studies, with an increase in the proportion of surfactant in the micellar system. Previous studies showed that high proportions of surfactant improve the wettability and dispersion of the particles in the dissolution medium [9].

### 3.7. Pharmacokinetic Study

EZ conjugates after oral administration to form the active glucuronide metabolite (EZ glucuronide). EZ-glucuronide represents 80%–90% of the total amount of EZ in plasma (total EZ), while only 10% or 20% is free EZ. EZ-glucuronide has been reported to be more effective than EZ [1,5,12]. Both drugs exhibit complex pharmacokinetic profiles mainly due to repetitive enterohepatic kinetics [1,5].

**Figure 6:**
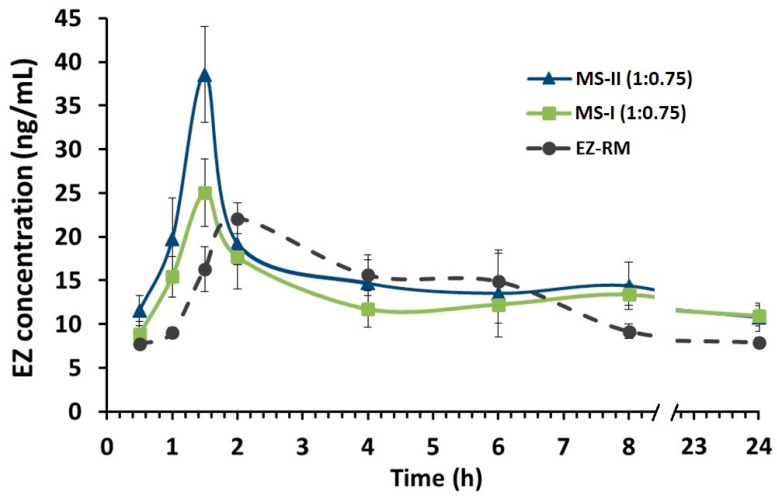
Plasma concentration profile of EZ raw material (EZ-RM), micellar system with Kolliphor^®^ RH40 (MS-I 1:0.75) and micellar system with Kolliphor^®^ RH40 (MS-II 1:0.75). Mean ± SD with n = 6 (EZ dose of 3.0 mg/kg).

EZ-glucuronide can be reformulated to EZ with the enzyme β-glucuronidase, which allows an analysis of all the EZ in blood (EZ-glucuronide and free-EZ) as total EZ [5]. To assess the improvement in pharmacokinetic behaviour, the plasma concentration-time curve profiles of total EZ after the oral administration of the optimized micellar system MS-II (1:0.75) were compared with EZ-RM and micellar system MS-I (1:0.75), as shown in Figure 6.

In this pharmacokinetic study, a direct relationship was observed between the increase in dissolution profiles and their pharmacokinetic profiles. MS-II (1:0.75) had the highest profiles, followed by MS-I (1:0.75) and EZ-RM, represented by a first time-point between one and two hours (Figure 6). Several studies report that the increase in the pharmacokinetic profile of poorly soluble drugs was due to different pharmaceutical systems that improve their solubility and dissolution rates [5,25].

Figure 6 also shows the second time-point of EZ-RM at six hours, whereas in MS-II (1:0.75) and MS-I (1:0.75) this second point occurred at eight hours. These multiple peaks are characteristic of the enterohepatic circulation of EZ formulations [1,12], and may be due to the EZ excreted into the bile after undergoing extensive glucuronidation to phenolic glucuronide in the intestine. The enhanced residence time via enterohepatic circulation increases its cholesterol-lowering activity [1].

The mean pharmacokinetic parameters (C_max_, T_max_ and AUC_0–24h_) for the three groups are summarized in Table 2. The data revealed significant improvements in the rate and extent of drug absorption from MS-II (1:0.75) and MS-I (1:0.75) compared to EZ-RM. The C_max_ of MS-II (1:0.75) was 156.02% and 173.86% compared to MS-I (1:0.75) and EZ-RM, respectively. MS-II (1:0.75) and MS-I (1:0.75) exhibited an earlier T_max_ (1.5 h) than EZ-RM (T_max_ 2 h). Previous studies with micellar systems showed increased plasma concentrations in the early stages [5].

The relative bioavailability of EZ in MS-II (1:0.75) was about 142.99% compared to EZ-RM, indicating rapid absorption and higher bioavailability of EZ from these micellar systems. The statistically significant (*p* < 0.001) higher bioavailability of EZ from MS-II (1:0.75) highlights the role of the fast dissolution profile in enhancing drug absorption. The slight improvement (114.24%) in relative bioavailability observed between MS-II (1:0.75) and MS-I (1:0.75) could be attributed to the smaller difference in early dissolution times between these systems. However, the mean C_max_ of MS-II (1:0.75) showed significant increases (*p* < 0.001) of 173.86% and 156.02% compared to EZ-RM and MS-I (1:0.75), respectively.

The interactions of MS-II (1:0.75) with biological fluids may vary due to differences in the preparation process of both types of micellar systems, which could account for the variation in C_max_ values between MS-II (1:0.75) and MS-I (1:0.75). Both EZ formulations have the same proportions of Kolliphor^®^ RH40; this amount of surfactant may produce a similar increase in permeability and inhibit the outflow of P-gp [13]. The differences between MS-II (1:0.75) and MS-I (1:0.75) have been attributed to a higher proportion of amorphous EZ in PXRD and DSC studies, which improved its dissolution.

An increase in the pharmacokinetic parameters of MS-II (1:0.75) could enhance cholesterol-lowering activity. Studies on the efficacy of MS-II (1:0.75) are important to evaluate the effect of this micellar system on total cholesterol and lipid parameters (TC, TG, LDL and HDL) compared to EZ-RM.

### 3.8. Biochemical Parameters

**Figure 7:**
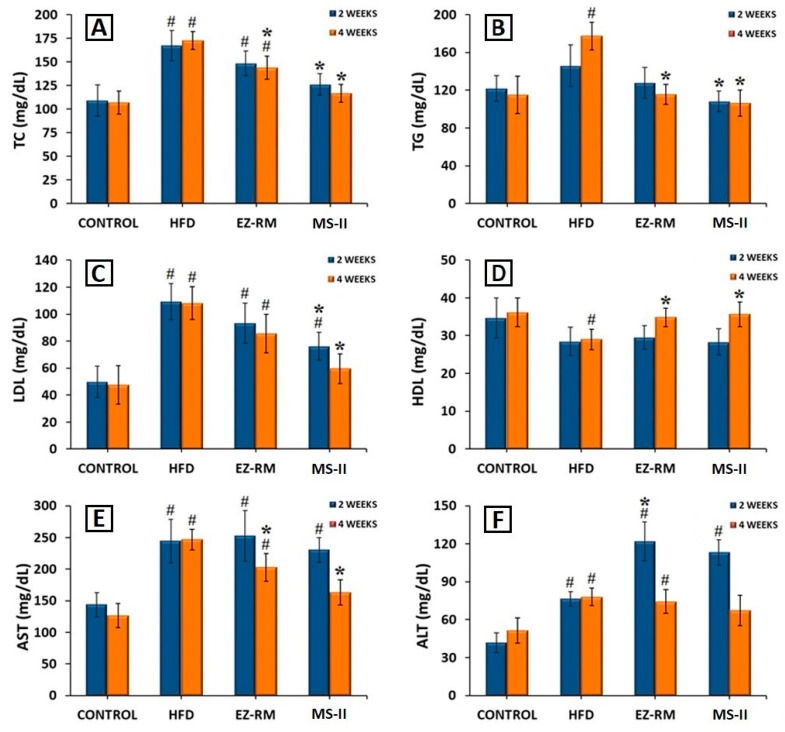
Serum levels of (**A**) total cholesterol (TC), (**B**) triglycerides (TG), (**C**) low-density lipoproteins (LDL), (**D**) high-Density lipoproteins (HDL), (**E**) aspartate transaminase (AST) and (**F**) alanine transaminase (ALT), after 2 and 4 weeks of treatment. Mean ± SD (n = 6) of the following groups: Control, high-fat diet (HFD), EZ raw material (EZ-RM) and micellar system with Kolliphor^®^ RH40 (MS-II 1:0.75). (#) significant difference (*p* < 0.05) compared with Control group; (*) significant difference (*p* < 0.05) compared with HFD group.

For this study, the rats in the HFD, EZ-RM and MS-II groups were fed a high-fat diet (HFD) to induce hyperlipidemia, while the animals in the control group were fed a standard diet. After two and four weeks of treatment, the EZ-RM and MS-II (1:0.75) groups exhibited significant changes in TC, TG, LDL, HDL, ALT and AST. All these values are shown in Figure 7.

After two weeks of treatment, the hyperlipidemia induced in the animals by the HFD produced an increase of 53.37% in TC, 19.53% in TG and 119.30% in LDL levels, and a reduction of 17.80% in HDL levels compared to the animals in the control group fed a standard diet (Figure 7A–D). AST and ALT levels also increased significantly (*p* < 0.05) by 69.82% and 82.88%, respectively, in comparison with the control group (Figure 7E,F). Previous work indicates that these high levels of TC and lipids are related to liver steatosis and inflammation, and are indicative of non-alcoholic fatty liver disease [26].

After two weeks of treatment, the animals in the EZ-RM group showed a slight improvement (*p* > 0.05) in TC, TG, LDL and HDL values compared to the HFD group. However, the animals treated with the MS-II (1:0.75) formulation presented a significant decrease (*p* < 0.05) in TC (24.53%), TG (25.71%) and LDL (30.44%) levels (Figure 7A–C) in comparison with the HFD group. The HDL values after two weeks of treatment with MS-II (1:0.75) were similar to those of the HFD group (Figure 7D). The decrease in LDL levels with the administration of MS-II (1:0.75) could improve the recovery time of the liver cells.

Transaminase levels showed no differences for AST values compared to the HFD group after two weeks of EZ-RM treatment, and only a slight decrease in AST was seen in the MS-II (1:0.75) group compared to the HFD group (Figure 7E). However, after two weeks of treatment the ALT values presented significant increases (*p* < 0.05) of 59.25% for the EZ-RM group and 48.14% for the MS-II (1:0.75) group (Figure 7F). The high values of ALT in the EZ treatment groups (EZ-RM and MS-II (1:0.75)) may be due to increased liver metabolism due to fat accumulation in the hepatocytes [27].

At four weeks the animals fed the high-fat diet (HFD group) presented similar values of TC, LDL, HDL, AST and ALT compared to the levels at two weeks, and only the TG parameters showed an increase of 21.60% (Figure 7B).

After four weeks of EZ-RM treatment, TC and TG levels showed a significant decrease (*p* < 0.05) of 16.80% and 34.59%, respectively (Figure 7A,B), and an increase (*p* < 0.05) in HDL levels of about 20.10% compared to the HFD group (Figure 7D). LDL values showed a slight decrease, with no significant differences with the HFD group (Figure 7C). These LDL and TC levels were significantly different from the control group. At this dose of ezetimibe (3 mg/kg/day), more prolonged treatment may be necessary to achieve lipid levels similar to those seen in the control group [28].

The serum analysis of the animals at the end of the treatment (four weeks) with MS-II (1:0.75) showed improvements of 32.53% for TC, 40.04% for TG, 44.95% for LDL and 23.00% for HDL compared to the HFD group (Figure 7A–D). These values presented no significant differences (*p* > 0.05) with the animals fed the standard diet (control group). However, the TC and LDL levels of the MS-II (1:0.75) group were significantly (*p* < 0.05) different from the EZ-RM group.

The higher plasma concentrations of EZ with MS-II (1:0.75) explain the greater TC and lipid reduction achieved with this treatment, due to the enhanced solubility of ezetimibe in this formulation. The decrease in the accumulation of TC, TG and LDL in the liver plays an important role in reducing hepatic steatosis [26].

The AST levels at four weeks of treatment showed a significant decrease (*p* > 0.05) of 17.83% with the EZ-RM treatment, and 33.89% with MS-II (1:0.75) compared to the HFD group (Figure 7E), significantly different (*p* > 0.05) from the control group. However, the AST values in the MS-II (1:0.75) group showed no significant differences (*p* < 0.05) with the control group. The EZ-RM and MS-II (1:0.75) groups presented a decrease of 4.71% and 13.89% in ALT values, respectively, compared to the HFD group (Figure 7F).

The lower ALT and AST levels in the EZ-RM and MS-II (1:0.75) groups could be due to a decrease in the hepatic cholesterol metabolism after four weeks of treatment [29]. The ALT levels of animals treated with the MS-II (1:0.75) formulation were not significantly different (*p* < 0.05) to those of the control group. This treatment had an advantage over EZ-RM due to the significant decrease in TC and lipid parameters, and its lower AST and ALT levels could imply fewer adverse effects on muscles and liver than in the EZ-RM and HFD groups [30].

These results demonstrate the importance of ALT and AST analysis to evaluate the metabolism of liver cells in patients with antihyperlipidemic treatment. Elevated transaminase levels at two weeks indicate higher liver metabolism, whereas decreases in ALT and AST values at four weeks of treatment could indicate lower metabolic activity due to a decrease in fat accumulation in hepatocytes.

## 4. Conclusions

In this study, two types of EZ micellar systems were prepared with different manufacturing processes. These formulations, MS-I and MS-II, with proportions of EZ:Kolliphor^®^ RH 40 of 1:0.25, 1:0.5 and 1:0.75, were developed to increase the solubility and dissolution rate of EZ.

PXRD and DSC studies showed a difference in the percentage of the amorphous form of EZ between MS-I (1:0.25) and MS-I (1:0.75), and an increase in the amorphous form of EZ in MS-II compared to MS-I. The process used to prepare MS-II with high ratios of surfactant (1:0.75), produced the highest amounts of the amorphous form of EZ.

The partial recrystallization of microcrystalline cellulose on the MS-I micellar systems that was observed in the PXRD, DSC and SEM studies was due to the delayed dissolution profiles of EZ in these formulations. The improvement in dissolution profiles for MS-II micellar systems compared to MS-I formulations was attributed to the differences in physicochemical characteristics in both types of micellar systems.

Pharmacokinetic studies of MS-II (1:0.75) showed a significant improvement (*p* < 0.05) for C_max_ and increased relative bioavailability compared to EZ-RM, indicating a possible relation between the higher dissolution profiles of EZ in MS-II and the greater bioavailability of these micellar systems.

The efficacy studies showed an improvement (*p* < 0.05) in TC, TG, LDL and HDL levels in the MS-II (1:0.75) group compared to the group fed a high-fat diet (HFD) in the absence of treatment. At the end of this study the MS-II (1:0.75) group also exhibited a significant decrease in TC and LDL levels compared to the EZ-RM group. The analyses of transaminase levels revealed decreases in ALT and AST at four weeks of treatment in the MS-II (1:0.75) group, compared to the EZ-RM group.

## Figures and Tables

**Table 1:** Flow properties of ezetimibe (EZ) raw material (EZ-RM), solid dispersion (SD), micellar systems with Kolliphor RH40^®^ MS-I (1:0.75) and MS-II (1:0.75) with different ratios of EZ/MC (*w/w*). Data are presented as mean ± standard deviation with n = 3.

	Ratio EZ/MC (*w/w*)	Angle of Repose (°)	Carr’s Index (%)	Hausner Ratio
EZ-RM	-	>40	35.51 ± 2.92	1.55 ± 0.07
SD	-	33.79 ± 1.38	33.02 ± 1.27	1.53 ± 0.03
MS (1:0.75) *	-	32.67 ± 1.24	33.45 ± 0.80	1.56 ± 0.02
SD	10/50	25.48 ± 1.21	28.77 ± 1.02	1.43 ± 0.04
MS-I (1:0.75) *	10/50	29.13 ± 1.29	30.47 ± 0.96	1.42 ± 0.02
MS-II (1:0.75) *	10/50	27.92 ± 0.71	29.99 ± 2.20	1.43 ± 0.05
SD	10/100	17.05 ± 0.95	18.26 ± 1.82	1.22 ± 0.03
MS-I (1:0.75) *	10/100	22.67 ± 2.31	20.14 ± 1.32	1.25 ± 0.02
MS-II (1:0.75) *	10/100	17.67 ± 2.08	17.55 ± 2.14	1.24 ± 0.03

* No significant differences (*p* > 0.05) were observed between MS-I and MS-II formulations with different EZ:Kolliphor^®^ RH40 ratios (1:0.25), (1:0.5) and (1:0.75).

**Table 2:** Pharmacokinetic parameters of EZ raw material (EZ-RM), micellar systems with Kolliphor RH40^®^ MS-I (1:0.75) and MS-II (1:0.75). Mean ± standard deviation with n = 6.

	EZ-RM	MS-I (1:0.75)	MS-II (1:0.75)
AUC (ng h/mL) ^a^	253.13 ± 17.90	299.55 ± 15.66	332.76 ± 32.26
C_max_ (ng/mL) ^b^	22.13 ± 1.86	24.66 ± 4.41	37.10 ± 7.88
T_max_ (h) ^c^	2.5 ± 1.20	1.50 ± 1.05	1.50 ± 1.04

^a^ Area under the drug concentration time curve. ^b^ Maximum plasma concentration of the drug. ^c^ Time taken to reach the maximum plasma concentration.

## Abbreviations

AUC	Area under curve
ALT	Alanine transaminase
AST	Aspartate transaminase
CI	Carr’s index
CrI	Crystallinity index
C_max_	Maximum plasma concentration
CPMP	Committee for Proprietary Medicinal Products
DSC	Differential scanning calorimetry
EZ	Ezetimibe
HDL	High-density lipoprotein
HFD	High-fat diet
HPC	Hydroxypropyl cellulose
HPLC	High-performance liquid chromatography
HPMC	Hydroxypropyl methylcellulose
ICH	International Conference on Harmonization
LDL	Low-density lipoprotein
MC	Microcrystalline cellulose
P-gp	P-glycoprotein
PM	Physical mixture
PXRD	Powder X-ray diffraction
RM	Raw material
SD	Solid dispersion
SEM	Scanning electron microscopy
MS	Micellar system
TC	Total colesterol
TG	Triglycerides
T_max_	Time of maximum plasma concentration
USP	United States Pharmacopeia
